# Community-acquired bacteremia and acute cholecystitis due to *Enterobacter cloacae:* a case report

**DOI:** 10.4076/1752-1947-3-7417

**Published:** 2009-09-11

**Authors:** Guillermo Isasti, Laura Mora, Victoria García, Jesus Santos, Rosario Palacios

**Affiliations:** 1 Internal Medicine Department, Hospital Virgen de la Victoria, Campus Teatinus s/n, 29010 Málaga, Spain; 2 Microbiology Department, Hospital Virgen de la Victoria, Campus Teatinus s/n, 29010 Málaga, Spain; 3 Infectious Diseases Unit, Hospital Virgen de la Victoria, Campus Teatinus s/n, 29010 Málaga, Spain

## Abstract

**Introduction:**

*Enterobacter cloacae* is responsible for 65-75% of all *Enterobacter* infections, bacteremia being the most common syndrome. The majority of infections are nosocomially acquired and in patients with predisposing factors.

**Case presentation:**

We present a case of *E. cloacae* bacteremia secondary to acute cholecystitis in a 60-year-old man with recent diagnosis of cholelithiasis. The diagnosis was established with abdominal echography and positive blood and biliary cultures. The patient was managed successfully with cholecystectomy and antibiotic therapy.

**Conclusion:**

The peculiarity of our case is the development of community-acquired bacteremia due to *E. cloacae* with a clear infectious focus, as a single agent isolated in several blood cultures, in a patient without severe underlying diseases, prior antimicrobial use or previous hospital admission. Although the majority of *Enterobacter* spp. infections are nosocomially acquired, primary bacteremia being the most common syndrome, these pathogens may also be responsible for community-acquired cases. Patients without predisposing factors may also be affected.

## Introduction

*Enterobacter* spp. have been recognized as increasingly important pathogens in recent years, and have been implicated in a broad range of clinical syndromes, the majority of which are nosocomially acquired and in patients with predisposing factors [1]. *Enterobacter cloacae* is responsible for 65-75% of all *Enterobacter* infections, bacteremia being the most common syndrome [1]-[5]. A patient with bacteremic acute cholecystitis is presented.

## Case presentation

A 60-year-old previously healthy man, with a recent diagnosis of cholelithiasis, was admitted to our hospital with a 24-hour history of high fever, nausea and severe pain in the right hypochondrium. On clinical examination, mild jaundice, abdominal tenderness in the right upper quadrant and a positive Murphy sign were noted. His blood pressure was 110/70 mmHg, his pulse was 130 beats per minute and his temperature was 39.5ºC. He had no leukocytosis but had significant neutrophilia, an elevated C reactive protein level, elevated erythrocyte sedimentation rate and elevated total bilirubin with an elevated direct fraction. An abdominal ultrasound revealed a relaxed gallbladder, with a thickened wall with multiple biliary calculi and biliary mud. Empirical treatment for acute cholecystitis was initiated with piperacillin-tazobactam 4.5 g every 8 hours, which is one of the options in our hospital's protocol for the treatment of biliary tract and gallbladder infections, mainly in very ill patients, and patients turned down for emergency surgery. Three days later, all sets of the blood cultures grew *E. cloacae*, which was sensitive to empirical antibiotics. In imaging techniques, such as computed tomography and magnetic cholangioresonance, a collection was observed in the anterior subhepatic space, suggesting an abscess. A cholecystectomy was therefore performed, obtaining biliary abscess exudate in which *E. cloacae* also grew, with the same susceptibility as *E. cloacae* isolated from blood cultures. The patient's condition had improved after starting antibiotic treatment, even before surgery, and no further bacteremia was detected. Piperacillin-tazobactam was maintained up to 4 weeks in addition to support treatment. The patient had a favorable outcome, leaving hospital 10 days later.

## Discussion

The genus *Enterobacter* belongs to the family *Enterobacteriaceae*; 14 species of this genus have been described, and *E. cloacae* and *E. aerogenes* are by far the most frequently encountered human pathogens among them [1]. Although community-acquired infections with *Enterobacter* spp. do occur, the majority of infections are nosocomial [1]-[6]. Patients at increased risk of acquiring an *Enterobacter* infection include those with a prolonged hospital stay, especially if a portion of it is spent in an intensive care unit [1,3]-[5]. The incidence of bacteremia has increased gradually accounting for nearly 11% of nosocomial infections in some series [6,7]. The condition for development of bacteremia due to *Enterobacter* spp. is severe underlying illness, and the most commonly cited factors associated with the acquisition of bacteremia due to *Enterobacter* spp*.* are neoplastic disease, diabetes mellitus, chronic renal failure, and gastrointestinal diseases [1,3]-[6]. Prior antimicrobial exposure, in particular, to penicillins, has also been identified as a risk factor for this entity [6]. Compared with bacteremias due to other enteric bacilli, *Enterobacter* spp. are acquired significantly more often in hospitals, are more likely to be a component of a polymicrobial bacteremia, and the portal of entry is usually unknown [1]-[5]. When the source of infection can be identified, the major portals of entry are the genitourinary and gastrointestinal tracts, followed by others such as intravascular catheters, the respiratory tract and surgical wounds [1,6]. The gastrointestinal tract is a common endogenous reservoir for *E. cloacae* and spread of infection from the gastrointestinal tract is difficult to ascertain, which can explain why the portal of entry often cannot be identified [6,7].

In our patient, bacteremia was associated with an acute cholecystitis complicated by the development of a subhepatic abscess that required surgery, and the same *E. cloacae* was isolated in both blood and abscess exudates. As far as we know, there has been no other reported case of acute bacteremic cholecystitis due to *E. cloacae* as a single etiologic agent. With regard to antimicrobial susceptibility, *E. cloacae* is resistant to ampicillin, cefalothin and other older cephalosporins, and cefoxitin. The carbapenems, cefepime, aminoglycosides, and ciprofloxacin are active against the majority of strains of *E. cloacae*[8]-[10].

## Conclusion

The peculiarity of our case is the development of community-acquired bacteremia due to *E. cloacae* with a clear infectious focus, an acute cholecystitis, as a single agent isolated in several blood cultures, in a patient without severe underlying diseases, prior antimicrobial use or previous hospital admission. As far as we know, there is no other reported similar case in patients without comorbidity.

Although the description of this case has little implication for conventional clinical practice, as blood cultures, antibiotherapy and surgery are usually included in cases similar to ours, it does highlight the possibility of a community-acquired bacteremia due to traditional nosocomial microorganisms in patients without severe underlying diseases, prior antimicrobial use or previous hospital admission.

## Consent

Written informed consent was obtained from the patient for publication of this case report. A copy of the written consent is available for review by the Editor-in-Chief of this journal.

## Competing interests

The authors declare that they have no competing interests.

## Authors' contributions

GI and RP: study concept and design, patient care, manuscript's draft and review, and final approval of the version to be published. LM and VG: microbiological procedures of the samples, and final approval of the version to be published. JS: contribution to study design and concept, critical review of the manuscript, and final approval of the version to be published.
